# The “one size fits all” approach to trauma treatment: should we be satisfied?

**DOI:** 10.3402/ejpt.v6.27344

**Published:** 2015-05-19

**Authors:** Marylene Cloitre

**Affiliations:** 1 National Center for PTSD Division of Dissemination and Training, Department of Psychiatry and Behavioral Sciences, Stanford University, Stanford, CA, USA; 2 Department of Psychiatry and of Child and Adolescent Psychiatry, NYU Langone Medical Center, New York, NY, USA

**Keywords:** PTSD, complex PTSD, patient preferences

## Abstract

There have been significant advances in the treatment of posttraumatic stress disorder in the last two decades. Further improvements in outcomes will be supported by recognition of the heterogeneity of symptoms in trauma populations and the development of treatments that promote the tailoring of interventions according to patient needs. Collaboration with patients regarding preferences about treatment structure, process, and outcomes is critical and will benefit the effectiveness and quality of treatments as well as the speed of their dissemination. New research methodologies are required that can incorporate important variables such as patient preferences and symptom heterogeneity without necessarily extending already lengthy study times or further complicating study designs. An example of alternative methodology is proposed.

Several treatment guidelines generally agree on recommending trauma-focused psychological therapies, specifically trauma-focused cognitive-behavioral therapy (TF-CBT), and Eye Movement Desensitization and Reprocessing (EMDR) as first-line treatments for posttraumatic stress disorder (PTSD) (e.g., National Institute for Clinical Excellence [NICE], 2005; Australian Centre for Posttraumatic Mental Health [ACPMH], 2007). However, these same guidelines identify caveats regarding implementing trauma-focused therapies for populations that have experienced complex trauma and who have more complex symptom profiles. The purpose of this article is to identify reasons for caution in taking a “one size fits all” approach to PTSD patients and to consider alternatives. First, the effects of trauma exposure are heterogeneous and this heterogeneity is not addressed in many of the evidence-based therapies available to date. It is important to maintain an attitude of curiosity and innovation toward developing new therapies or adapting current therapies that recognize the presence of distinct symptom profiles. Second, some scientific inquiries have proceeded with the assumptions that “briefer is better” and that confronting the traumatic past is a critical first step. Recent research indicates that trauma-focused work may not be a necessary element in effective treatment nor that it be the first step required in effective trauma recovery. Finally, PTSD treatment development and implementation has occurred without the substantial involvement of patients and trauma survivors, who have particular insight about their primary concerns and treatment preferences. Although PTSD is the primary outcome in most clinical trials, the assumption that it is the outcome of primary interest to traumatized individuals or communities has not been evaluated. Collaboration with patients regarding preferences about treatment structure, process, and outcomes will benefit the effectiveness and quality of treatments as well as the speed of their dissemination.

## Different patient populations

The development of diverse treatments is merited only if there are subgroups of trauma populations that differ from one another in important ways. There is substantial evidence for this. Several research studies have found that an increasing number of different types of trauma (trauma complexity) is associated with an increasing number of different types of symptoms beyond PTSD (symptom complexity). Typically, these include emotion regulation difficulties, interpersonal difficulties, substance abuse problems, anger, dissociation, and suicidality. The association between trauma and symptom complexity has been found in epidemiological studies (Karam et al., 2014), community samples (Briere, Kaltman, & Green, 2008), and clinical samples of both adults and children (Cloitre et al., 2009). Experience of childhood complex trauma is a notable adversity. Cloitre et al. (2009) found that although both adulthood and childhood experiences of complex trauma predicted symptom complexity, cumulative trauma during childhood was by far the stronger contributor.

### ICD-11

Related research has evaluated symptom complexity using categorical analyses as exampled by the newly proposed World Health Organization (WHO) ICD-11 diagnoses of PTSD and Complex PTSD (CPTSD) (Maercker et al., 2013). In this formulation, the symptoms of PTSD directly result from stimuli related to the traumatic events (re-experiencing, avoidance, and the consequent hyperarousal) whereas CPTSD includes the PTSD symptoms as well as three additional clusters that reflect the types of disturbances trauma can have on systems of self-organization, specifically in affective, self-concept, and relational domains, and are problems that are more typically the consequence of sustained, repeated, or complex trauma.

Latent class analyses have indicated that trauma survivors fall into groups consistent with the distinct symptom profiles associated with the PTSD versus CPTSD diagnoses. The ICD-11 PTSD versus CPTSD distinction has been supported in six studies to date which include assessment in quite different samples including those experiencing a range of interpersonal violence events (Cloitre, Garvert, Brewin, Bryant, & Maercker, 2013), rape victims, survivors of domestic violence and of traumatic bereavement (Elklit, Hyland, & Shevlin, 2014), victims of institutional abuse such as that occurring within foster care and religious organizations (Knefel, Garvert, Cloitre, & Lueger-Schuster, 2015), as well as community samples of veterans (Wolf et al., 2014) and young adults (Perkonigg, Höfler, Wittchen, Trautmann, & Maercker, 2014). Preliminary data indicate the distinction is observed among clinical samples of trauma-exposed children (Stolbach, Garvert, & Cloitre, 2014). In addition, analyses indicate that CPTSD is more commonly found among those with complex trauma histories, such as childhood abuse, is associated with greater impairment (Cloitre et al., 2013; Perkonigg et al., 2014), and can be distinguished from Borderline Personality Disorder (Cloitre, Garvert, Weiss, Carlson, & Bryant, 2014).

### DSM-5

The DSM-5 recognizes the presence of emotion regulation, self and interpersonal disturbances, many of which are similar to those identified in ICD-11 Complex PTSD. For example, persistent negative beliefs about oneself are now included in a new cluster called “negative alterations in cognitions and mood” whereas emotion dysregulation as expressed in aggressive, reckless, or self-destructive behavior is incorporated in a cluster described as “alterations in arousal and reactivity.” In addition, a dissociative subtype has been added to the disorder composed of symptoms of derealization and/or depersonalization (see Friedman, 2013).

Perhaps the greatest difference between ICD-11 and DSM-5 is that in DSM-5 all of the symptoms are included under a single “big tent” or “broad definition” of PTSD whereas ICD-11 presents two “sibling” diagnoses. The DSM-5 decision was in part driven by DSM guidelines in which an existing diagnosis was to be maintained unless there was strong scientific evidence to modify the disorder (Friedman, 2013). Thus, new symptoms that had strong empirical evidence as being a consequence of trauma were included in the diagnosis, but their inclusion was not viewed as demanding an alteration in the structure of the diagnostic taxonomy.

This symptom-inclusive DSM-5 formulation has been described as resulting in an excessively large number of mathematically possible ways by which individuals can be diagnosed with PTSD (Galatzer-Levy & Bryant, 2013), leading to the concern that the number of different symptom profiles that can emerge under diagnosis are heterogeneous beyond clinical value. Practically speaking, the same label might be applied to patients with very different symptom profiles, reducing the usefulness of the label as a means of communication among clinicians and patients. Moreover, given the addition of several symptoms and the re-organization of several symptom clusters, it is fair to ask whether the treatments developed for the old DSM-IV diagnosis work as well for the new version.

The WHO guidelines for the development for ICD-11 and the taxonomic structure of the ICD-11 diagnoses are quite different from those for DMS-5. WHO guidelines recommended that the guiding principle in the revisions of disorders follow the potential clinical utility of the formulation (Reed, 2010). In addition, the taxonomic structure of the ICD-11 is organized such that it supports the presence of “sibling” disorders (e.g., PTSD and CPTSD) under a larger “parent” category (e.g., posttraumatic stress disorders), an option that does not exist in DSM. The emphasis on clinical utility meant that the formulation of the diagnoses should be consistent with typical ways in which clinicians organize information, that the symptom patterns presented in the diagnoses should be easily discernible and distinguishable from one another, and that the diagnoses be transparently related to a treatment plan. As described earlier, studies thus far using archival data have demonstrated the construct validity of the PTSD/Complex PTSD distinction. In addition, there is emerging evidence of the clinical utility of this distinction. The results of the ICD-11 field trials in which approximately 1,500 clinicians across the globe were presented with case scenarios have revealed that clinicians consistently perceived, distinguished, and accurately identified ICD-11 PTSD versus CPTSD cases (Keeley, 2014).

### Future research

A critical task in the immediate future is to develop and test interview and self-report measures of the ICD-11, PTSD, and CPTSD diagnoses (Bisson, 2014). Although analyses of archival data approximating these constructs has been an important first step in supporting the construct validity and discriminability of PTSD and CPTSD, the development of reliable measures is a prerequisite for further scientific inquiry. Studies directly comparing the presence of DSM-5 as compared to ICD-11 diagnoses in large clinical and epidemiological samples would help determine the extent to which the diagnoses capture same or different populations. Research investigating the presence of neurobiological differences that distinguish the trauma-related symptom profiles would contribute to basic science and provide a neurobiological foundation for observed symptom profiles. Last, studies investigating the clinical utility for both ICD-11 and DSM-5 should be assessed and compared particularly in relation to facilitating treatment planning, selecting treatments, and enhancing patient outcomes.

## Optimal treatment outcomes: are we there yet?

Trauma-focused treatments typically include repeated *in vivo* and/or imaginal exposure to the trauma, reappraisal of the meaning of the trauma and its consequences (cognitive interventions), or some combination of these techniques. These therapies have been identified as efficacious for a range of PTSD sufferers, including rape victims, survivors of childhood abuse, refugees, combat veterans, and victims of motor vehicle accidents (Foa, Keane, Friedman, & Cohen, 2009). Nevertheless, meta-analyses reveal that approximately 40% of treatment completers maintain their PTSD diagnoses after TF-CBTs, and even among those who no longer have PTSD, the majority still suffer from significant residual symptoms (Bradley, Greene, Russ, Dutra, & Westen, 2005). These data overall suggest that there is room, perhaps even urgent need, for improvement in our therapies for trauma populations.

### Differential responses based on patient characteristics

One approach to reaching the above goal is to identify subgroups of patients who appear to obtain relatively less benefit in TF-CBTs and adapt treatments accordingly. However, studies attempting to identify patient characteristics that predict differential outcome are limited in number and have produced inconsistent results. One reason may be the substantial heterogeneity of symptoms in trauma populations and the lack of a consistently articulated characterization of distinct subgroups of PTSD patients. This circumstance may resolve in time with the emergence of a stable and reliable categorization plan (e.g., ICD-11 PTSD versus CPTSD). In the absence of anything more substantial, predictor studies to date have relied on the examination of individual patient characteristics in a one-by-one fashion to identify potential risk factors for poorer outcome. Using this approach, some studies have found that typical comorbidities such as depression, anger, dissociation, and shame predicted less good outcome (Taylor et al., 2001; Van Minnen, Arntz, & Keijsers, 2002; Hagenaars, Van Minnen, & Hoogduin, 2010) but this has not always been found in other studies (Rizvi, Vogt, & Resick, 2009) or even in the same study and same intervention but with a different patient population (Van Minnen et al., 2002). Better characterization of patient profiles and potentially, better methodologies for identifying treatment moderators are needed (see Wallace, Frank, & Kraemer, 2013).

### Different responses based on different therapies

A second approach to improving therapy outcomes has been to develop and test adaptations or extensions of trauma-focused treatments to enhance outcomes for identified vulnerable populations. These adaptations have most typically been evaluated among individuals with a history of childhood abuse. In the absence of a reliable measure of Complex PTSD, a history of childhood abuse, at least where the experience is prolonged and repeated, provides a reasonable proxy for complex symptom profiles. In addition, childhood abuse is of interest as it may have unique features specific to the impact of trauma on key developmental tasks as indicated in the developmental neurobiology and psychosocial literature (Heim & Nemeroff, 2001; Shipman et al., 2007).

The most recent meta-analysis of psychological treatments for PTSD among adult survivors of childhood sexual abuse identified 16 randomized clinical trials for review (Ehring et al., 2014). The results showed that TF-CBT, not including EMDR, yielded a larger pre-post effect size (Hedges’ g) than non-trauma-focused therapies (ES=1.34 vs. ES=0.82, respectively). A careful review of the eight trauma-focused CBT protocols that included individual therapy indicated that four were sequenced, multicomponent (Cloitre, Koenen, Cohen, & Han, 2002; Chard, 2005; Cloitre et al., 2010; Bohus et al., 2013) and four were purely trauma-focused treatments (McDonagh et al., 2005; Resick et al., 2008, which included three active treatments). The effect sizes for the former ranged from 2.27 to 1.31 whereas those for the purely trauma-focused treatment ranged from 1.37 to 0.70. A rank ordering of the effect sizes for the eight studies indicated that three of the four largest effect sizes (top half) were from the sequenced, multicomponent therapies. The use of EMDR therapy which includes a focus on traumatic memories as well as a preparation phase and multiple supporting interventions (e.g., positive images) (see Shapiro & Laliotis, 2015) was reported to substantially benefit childhood sexual abuse participants, yielding large pre-post effect sizes for PTSD symptoms ranging from 1.93 to 1.46 (Ehring et al., 2014).

The small number of studies does not allow any type of statistical analysis. In addition, one study (Chard, 2005) might not be considered “sequential” by some as only 5 weeks of intervention were provided before exposure therapy started (in individual therapy). On the other hand, in addition to the 5 weeks of preparation, participants received additional interventions preceding, co-occurring with, and extending beyond the exposure work which were of particular relevance to childhood trauma. These included education about the developmental impact of trauma and skills training in areas problematic for this population including assertiveness training, and sessions on social support and sexual intimacy. Even with these caveats, the studies overall suggest benefits and potential superiority of sequential and/or multicomponent therapies for those with PTSD related to childhood sexual abuse.

### Trauma-focused or not?

A final observation is the large effect sizes that are often obtained in non-trauma-focused therapies (see Bisson, Roberts, Cooper, & Lewis, 2013). Three meta-analyses have identified PTSD symptom effect sizes for non-trauma-focused treatments ranging from 0.40 to 0.82 (Dorrepaal et al., 2014; Ehring et al., 2014; Frost, Laska, & Wampold, 2014). These are moderate to large effect sizes indicating substantial observable change in PTSD symptoms. In addition, a recent study found that interpersonal therapy (IPT) was not inferior to prolonged exposure (Markowitz et al., 2015). This study is important as it is the first to report equivalence of outcomes when conducting a head-to-head comparison of a non-trauma-focused treatment IPT to a trauma-focused intervention.

### What is necessary versus what is optimal?

Some characterizations of the sequential, multicomponent therapy model have implied that such sequencing is viewed as *necessary* for PTSD recovery in those with complex forms of PTSD (Van Minnen, Harned, Zoellner, & Mills, 2012; De Jongh & Ten Broeke, 2014). The position is more accurately described as one which proposes that such therapies are likely to provide greater benefits relative to therapies comprised predominantly of trauma-focused interventions. Expert consensus opinion converges on this view (Cloitre et al., 2011) and treatment studies, as described above, are accumulating to support this position. In contrast, some other researchers have dismissed the importance of additions to trauma-focused therapies, recommended the delivery of exposure therapy with a variety of comorbidities, and proposed that delay in confronting traumatic memories with add-on interventions reduces the efficacy of the treatments by encouraging avoidance of the trauma (Van Minnen et al., 2012). The presence of such polarized views, in and of itself, suggests the importance of sustained research in comparing different types of available treatment interventions and in developing novel, alternative therapies that might resolve the tensions articulated above. Certainly, the results of the study by Markowitz and colleagues (2015) provoke a broader consideration: are trauma-focused interventions, the “core” or gold standard intervention for PTSD, *necessary* for the recovery of PTSD? The answer seems to be “no.” A more nuanced view of the treatment outcome literature would lead to the logical conclusion that no one type of treatment is necessary.

The primary question for current treatment research is: what therapies are optimal for which patients. Treatment research in physical diseases, particularly those that are highly heterogeneous, has indicated that optimization of treatment outcomes are related to personalized treatments that are tailored to variations in symptom profiles and that take into account patient preferences (Institute of Medicine, 2011). It would seem unlikely that similar advantages would not accrue by using such strategies for the treatment of PTSD and other mental health disorders.

A critical goal of good treatment care is to provide patients with a range of effective treatments from which to choose. This includes consideration about which problems they wish to address and in what order and, importantly, whether or not trauma-focused work is worth the result. Clearly, some patients have determined that they experience exposure therapy as too aversive relative to its benefits (see Morris, 2015). This experience and perspective should be respected and provide motivation among professionals to develop and provide alternative effective treatments for PTSD. The interpretation that patients are engaging in avoidance when they decline exposure therapies may be an overgeneralization, shutting out other considerations that relate to patient goals, values, and quality of life decisions, and ignores the inherent freedom of the patient to choose their own pace of recovery. The role of patient preference as a factor in the selection of a treatment and its influence on outcome via the mediating effect of variables, such as patient motivation or consistency of treatment with the patient's life goals and values, has yet to be studied but is emerging as an important research topic.

### Future directions

The evidence to date indicates that TF-CBTs benefit a range of trauma survivors, including those with more complex presentations. There is a limited but growing evidence base that enhancements to trauma-focused therapies provide superior outcomes. Much more research is necessary to identify optimal treatments for diverse trauma populations. A prerequisite to reaching this goal is to identify stable and reliable symptom profiles that predict differential outcome and can be used to match patients to the treatments that benefit them most. Head-to-head comparisons are necessary to determine whether or not sequential and/or multicomponent therapies provide clinically and statistically superior benefits compared to pure trauma-focused therapies on a range of outcomes among different types of complex trauma samples. In addition, provocative data suggest the importance of considering alternative interventions that may be neither trauma-focused nor multicomponent. Investigation of these types of interventions may lead to the availability of additional treatment choices for patients and may expand our knowledge of underlying mechanisms of recovery. Linkage of complex trauma profiles across the developmental years (See Ford, this issue) into adulthood and indeed through old age will promote understanding of stable versus changing core symptoms and the development of interventions relevant to the changing needs and preferences of patients in different phases of life.

The traditional randomized controlled trial design is not a particularly satisfactory methodology by which to identify optimal therapies for different and diverse patient populations. Patients (see Spinazzola, Blaustein, & Van der Kolk, 2005), therapists, settings, delivery, and duration of the treatment may be significantly different from that which is typical in the community. Effectiveness trials are a preferable starting point as they include the full range of trauma patients that clinicians must treat, typically are implemented by community clinicians, and are delivered taking into account “real world” factors such as limited staffing, “no-show” patients, and relapses into other disorders (e.g., substance abuse), leading to greater research-to-practice generalizability.

In addition, study designs that recognized and measured the impact of contextual factors in treatment delivery will support the successful implementation of manualized evidence-based treatments and help provide a more sophisticated understanding of when “treatment intervention” or therapeutic work truly begins. The newly emerging discipline of implementation science emphasizes the need to evaluate the impact of the therapeutic environment in which a clinical trial is embedded. Some researchers have reported successful work with trauma-focused therapies but not regarded the treatment context as supporting a level of functioning that is desirable for optimizing the outcome of trauma-focused work. For example, programs for highly traumatized and highly symptomatic patients (e.g., residential treatment programs, prisons) typically provide stable sleeping quarters, regular meals, structure to the day, a relatively high level of physical safety, medications (both psychotropic and for chronic illnesses), and several other psychosocial interventions that may support the trauma-focused work. Clinical trials that occur in outpatient or other settings in which there is ongoing “treatment as usual” may also provide unidentified but nevertheless significant contributors to good outcome. Identification of the components of care that precede or coexist with the delivery of PTSD manualized treatments is necessary to assess their contribution to overall improvement and to identify the level of resources needed to produce good outcome when PTSD treatments are introduced into new settings. Finally, patient preferences are a critical ingredient in every step of the treatment process and their incorporation will enhance engagement, adherence, and outcomes in therapy.

## Patient preferences

The recent mandate for patient-centered care in the United States, associated with the Patient Protection and Affordable Care Act of 2010, has articulated that patient-centered care requires the identification of outcomes about which patients care, the development of treatments that address these concerns, and research which routinely includes patient preference as a relevant factor in the selection of the optimal treatment. Recent data indicate that in the United States’ largest health care system, the Veteran's Affairs care system, where evidence-based trauma-focused therapies for PTSD have been well-disseminated, only about 10% of PTSD patients receive such treatments (Watts et al., 2014). Although there are system-related factors to consider (e.g., access to specialty PTSD services), important questions are whether patients are attracted to the treatments and more broadly how to improve patient engagement.

There has been some discussion as to whether incorporating motivational interviewing specific to increasing engagement in trauma-focused therapy will do the job (Slagle & Gray, 2007). Another alternative is to engage patients with interventions that appeal to them. Directly addressing presenting concerns other than PTSD and doing a good job of improving functioning and quality of life have both been an important impetus for the development of sequential modular treatment. Multipurpose engagement and rehabilitative interventions can include psychoeducation groups, emotion regulation and social skills training, yoga or exercise. Similarly, the use of m-health technologies may be an approach to engage patients who are unwilling or unable to make face-to-face visits (see Olff, this issue).

Ultimately, however, the essential task required in improving patient engagement is: asking the patient! Surveys of patient concerns are limited and those identifying patient preferences about treatment are fewer still. Research identifying patient psychosocial needs and treatment preferences is a prerequisite for developing true “patient-centered” care.

## Future directions in treatment research: patient-treatment matching

Ultimately, the ideal scenario would be to provide “personalized” care that is tailored to the individual which addresses problems that concern him or her most. Although this sounds like something that can only be realized in the distant future, there are patient-treatment matching models that have been tested, particularly for childhood disorders and which have been found to be successful. Psychological problems and disorders that occur during childhood, like those experienced by trauma populations, are highly heterogeneous. In recognition of this, child psychology and psychiatry researchers have proposed and tested a patient-treatment matching approach that orders treatment modules or interventions as they relate to a collaboratively identified hierarchy of problems. For example, a child with school phobia, depression, and conduct problems would complete an assessment where the importance of the problems would be collaboratively rank-ordered yielding a treatment plan where relatively specific, evidence-based interventions are matched to the hierarchy of problems. A resulting treatment plan might include a graded exposure hierarchy for school attendance, followed by mood-boosting activities for depression, and both of these implemented in tandem with a parent-driven incentives program to manage the child's conduct. These multicomponent intervention treatments have been found superior to the use of full protocols for a single disorder (Daleiden, Chorpita, Donkervoet, Arensdorf, & Brogran, 2006) or to the sequencing of full protocols for different disorders (Weisz et al., 2012).

The adaptation of this type of model is an important next step in treatment research concerning trauma-exposed populations. In this methodological approach, all interventions that have been previously tested and found effective are considered. These might include, for example, emotion regulation strategies (focused breathing, mindfulness, positive imagery) cognitive re-appraisal, body-based interventions (exercise, yoga), and/or meaning-making of the trauma (exposure, narratives, cognitive processing). The challenge in this effort is the preliminary work entailed in identifying interventions which has proven to be most effective across evidence-based protocols for the same and even for different disorders so that a maximally effective set of interventions is available for addressing specific problems. Once this is done, therapist and patient have a “toolkit” of interventions from which to choose. Although challenging to develop and test, this approach emphasizes collaboration between patient and therapist, supports patient preference in using interventions of their choosing, and holds the promise of relatively rapid relief through the use of evidence-based interventions effective for specific problems.

## Conclusion

The above summary suggests that a “one size fits all” approach to psychotherapy is inconsistent with the goals of maximizing patient outcomes and quality of care. The essay highlights the complexity of trauma, the heterogeneity of symptoms, and the importance of treatments tailored to the individual. Data is emerging that there are distinct and distinguishable groups of trauma patient populations and that sequential and/or multicomponent therapies may be superior for complex forms of PTSD. The article notes the lack of consideration of patient preference and calls for research evaluating the role of patient preference in enhancing treatment engagement, adherence, and outcome. A research model where patients are matched to interventions strategies is presented as a feasible and important future direction.

## Supplementary Material

The “one size fits all” approach to trauma treatment: should we be satisfied?

The “one size fits all” approach to trauma treatment: should we be satisfied?

The “one size fits all” approach to trauma treatment: should we be satisfied?

The “one size fits all” approach to trauma treatment: should we be satisfied?

The “one size fits all” approach to trauma treatment: should we be satisfied?

The “one size fits all” approach to trauma treatment: should we be satisfied?
