# Supplementary material for: The “one size fits all” approach to trauma treatment: should we be satisfied?
Source: Eur J Psychotraumatol. 2015 May 19;6:10.3402/ejpt.v6.27344. doi: 10.3402/ejpt.v6.27344 (PMC4439409; doi:10.3402/ejpt.v6.27344)
Supplement: The “one size fits all” approach to trauma treatment: should we be satisfied? [file EJPT-6-27344-s001.pdf]

L'approche "One Size Fits All" pour le traitement des traumatismes: Devrions-nous nous en satisfaire?

Marylene Cloitre

Il y a eu des progrès significatifs dans le traitement de l'état de stress post-traumatique (ESPT) lors des deux dernières décennies. D'autres améliorations dans les résultats sont susceptibles supportés par la reconnaissance de l'hétérogénéité des symptômes dans les populations traumatisées et le développement de traitements qui favorisent l'adaptation des interventions en fonction des besoins des patients. Une collaboration des patients concernant les préférences sur la structure de traitement, le processus et les résultats est essentielle et bénéficiera de l'efficacité et de la qualité des traitements ainsi que de la vitesse de leur diffusion. De nouvelles méthodologies de recherche sont nécessaires pouvant incorporer des variables importantes telles que les préférences des patients et l'hétérogénéité des symptômes sans étendre nécessairement des périodes d'études déjà longues ou de compliquer davantage les plans d'étude. Un exemple de méthodologie alternative est proposé.

Mots-clés: ESPT, ESPT complexe, préférences du patient

Citation: European Journal of Psychotraumatology 2015, **6**: 27344 - http://dx.doi.org/10.3402/ejpt.v6.27344
