# Supplementary material for: The “one size fits all” approach to trauma treatment: should we be satisfied?
Source: Eur J Psychotraumatol. 2015 May 19;6:10.3402/ejpt.v6.27344. doi: 10.3402/ejpt.v6.27344 (PMC4439409; doi:10.3402/ejpt.v6.27344)
Supplement: The “one size fits all” approach to trauma treatment: should we be satisfied? [file EJPT-6-27344-s002.pdf]

Der "eine Größe passt für Alle" Ansatz in der Trauma-Behandlung: Sollten wir zufrieden sein?

Marylene Cloitre

Es gibt signifikante Verbesserungen in der Behandlung der Posttraumatischen Belastungsstörungen (PTBS) seit den letzten zwei Jahrzehnten. Weitere Verbesserungen sind wahrscheinlich durch die Unterstützung und Anerkennung der Heterogenität der Symptome in von Traumatisierung betroffenen Bevölkerungsgruppen, ebenso durch die Entwicklung von maßgeschneiderten Interventionen, die auf die Bedürfnisse von Patienten Bezug nehmen. Ausschlaggebend könnte eine Zusammenarbeit mit Patienten hinsichtlich deren Präferenzen über Behandlungsstrukturen und Prozess, wodurch die Effektivität und die Qualität der Behandlung steigen könnten und ihre Verbreitung sich beschleunigen könnte. Neue Forschungsmethoden sind erforderlich, die so wichtige Variablen wie Patienten-Präferenzen und die Symptom-Heterogenität berücksichtigen, ohne dabei ohnehin lange Studiendauern zu verlängern oder das Studiendesign zu verkomplizieren. Eine alternative Methode wird vorgestellt.

Schlüsselwörter: PTBS, Komplexe PTBSD, Patienten-Präferenz

Citation: European Journal of Psychotraumatology 2015, **6**: 27344 - http://dx.doi.org/10.3402/ejpt.v6.27344
