# Supplementary material for: The “one size fits all” approach to trauma treatment: should we be satisfied?
Source: Eur J Psychotraumatol. 2015 May 19;6:10.3402/ejpt.v6.27344. doi: 10.3402/ejpt.v6.27344 (PMC4439409; doi:10.3402/ejpt.v6.27344)
Supplement: The “one size fits all” approach to trauma treatment: should we be satisfied? [file EJPT-6-27344-s003.pdf]

A „mind egy kaptafára" megközelítés a trauma terápiában: Meg vagyunk elégedve?

Marylene Cloitre

Jelentős előrelépés volt a poszttraumás stressz zavar (PTSD) kezelésében az elmúlt két évtizedben. További előrelépés várható, ha a különböző populációk tüneti sokszínűségét felismerjük és személyre szabott intervencióval tudunk szolgálni. A beteggel való együttműködés a terápiás preferenciáinak kialakításában (struktúra, folyamat) nagyon fontos a hatékony, minőségi és gyorsan disszeminálható intervenció kialakításában. Új kutatási módszerek szükségesek a beteg preferencia és tüneti heterogenitás figyelembevételére, anélkül hogy szükségtelenül meghosszabbítanánk a kutatások hosszát vagy bonyolítanánk a kutatási elrendezésen. Egy lehetséges példa kerül bemutatásra.

Kulcsszavak: PTSD; komplex PTSD, beteg preferencia

Citation: European Journal of Psychotraumatology 2015, **6**: 27344 - http://dx.doi.org/10.3402/ejpt.v6.27344
