# Supplementary material for: The “one size fits all” approach to trauma treatment: should we be satisfied?
Source: Eur J Psychotraumatol. 2015 May 19;6:10.3402/ejpt.v6.27344. doi: 10.3402/ejpt.v6.27344 (PMC4439409; doi:10.3402/ejpt.v6.27344)
Supplement: The “one size fits all” approach to trauma treatment: should we be satisfied? [file EJPT-6-27344-s004.pdf]

El enfoque "talla única" del tratamiento del trauma: ¿Deberíamos estar satisfechos?

Marylene Cloitre

Han habido avances importantes en las últimas dos décadas en el tratamiento del trastorno de estrés postraumático (TEPT). Es probable que se favorezca la mejoría en los resultados reconociendo la heterogeneidad de los síntomas en las poblaciones de trauma y desarrollando tratamientos que fomenten la creación de intervenciones en función de las necesidades del paciente. Es fundamental la colaboración con los pacientes en cuanto a las preferencias sobre la estructura, los procesos y los resultados del tratamiento y esto beneficiará la efectividad y la calidad de los tratamientos así como su velocidad de difusión. Se requieren nuevas metodologías de investigación que puedan incorporar variables importantes como preferencias del paciente y heterogeneidad de los síntomas, sin extender necesariamente los tiempos de estudio, que ya son largos, ni complicar aún más los diseños de los estudios. Ponemos un ejemplo de metodología alternativa.

Palabras clave: TEPT, TEPT complejo, preferencias del paciente

Citation: European Journal of Psychotraumatology 2015, **6**: 27344 - http://dx.doi.org/10.3402/ejpt.v6.27344
