# Supplementary material for: The “one size fits all” approach to trauma treatment: should we be satisfied?
Source: Eur J Psychotraumatol. 2015 May 19;6:10.3402/ejpt.v6.27344. doi: 10.3402/ejpt.v6.27344 (PMC4439409; doi:10.3402/ejpt.v6.27344)
Supplement: The “one size fits all” approach to trauma treatment: should we be satisfied? [file EJPT-6-27344-s005.pdf]

Travma Tedavisinde "Herkese Uyan" Yaklaşım: Memnun Olmalı mıyız?

Marylene Cloitre

Travma Sonrası Stres Bozukluğunun (TSSB) tedavisinde son 20 yıl içinde önemli gelişmeler olmuştur. Sonuçlardaki gelecek gelişimler travma popülasyonlarındaki semptomların çeşitliliğinin tanınması ve hastaların ihtiyaçlarına göre müdahalelerin biçimlendirilmesini destekleyen tedavilerin gelişimi ile desteklenebilir. Hastaların tedavi yapısı, süreci ve sonuçlarla ilgili tercihlerine göre hastalarla yapılan işbirliği oldukça önemlidir ve tedavinin etkisi ve kalitesi, aynı zamanda etkisinin yayılması, için yararlı olacaktır. Zaten uzun olan çalışma zamanını uzatmadan ve gelecek çalışma dizaynlarını karmaşıklaştırmadan semptom çeşitliliği ve hasta tercihleri gibi değişkenleri birleştirebilecek yeni araştırma yöntemlerine ihtiyaç duyulmaktadır.

Anahtar Kelimeler: TSSB, Karışık TSSB, Hasta Tercihleri

Citation: European Journal of Psychotraumatology 2015, **6**: 27344 - http://dx.doi.org/10.3402/ejpt.v6.27344
