# Supplementary material for: The “one size fits all” approach to trauma treatment: should we be satisfied?
Source: Eur J Psychotraumatol. 2015 May 19;6:10.3402/ejpt.v6.27344. doi: 10.3402/ejpt.v6.27344 (PMC4439409; doi:10.3402/ejpt.v6.27344)
Supplement: The “one size fits all” approach to trauma treatment: should we be satisfied? [file EJPT-6-27344-s006.pdf]

“Jedna veličina odgovara svima” pristup tretmanu traume: da li trebamo biti zadovoljni?

Marylene Cloitre

Došlo je do značajnog napretka u tretmanu posttraumatskog stresnog poremećaja (PTSP) u poslednje dve decenije. Dalji napredak ishoda lečenja biće podržan prepoznavanjem heterogenosti simptoma u populaciji osoba koje su preživele trauma i razvojem tretmana koji će promovisati prilagođavanje tretmana specifičnim potrebama pojedinca. Saradnja sa pacijentima u pogledu izbora prioritetne strukture tretmana, samog procesa i ishoda je ključan i povećaće efikasnost i kvalitet tretmana kao i brzinu njihove diseminacije. Potrebne su nove istraživačke metodologije koje bi uzele u obzir značajne varijable kao što su pacijentovi prioriteti i heterogenost simptoma bez nepotrebnog produžavanja vremena trajanja studija ili daljeg komplikovanja dizajna studija. Prikazan je primer alternativne metodologije.

Ključne reči: PTSP; kompleksni PTSP; pacijentovi prioriteti

Citation: European Journal of Psychotraumatology 2015, **6**: 27344 - http://dx.doi.org/10.3402/ejpt.v6.27344
